# Supplementary material for: Anillin directly crosslinks microtubules with actin filaments
Source: EMBO J. 2025 Jul 21;44(17):4803–24. doi: 10.1038/s44318-025-00492-3 (PMC12402178; doi:10.1038/s44318-025-00492-3)
Supplement: Supplementary file 1 — Appendix [file 44318_2025_492_MOESM1_ESM.pdf]

Appendix

Table of Contents

Appendix figure S1: Anillin forms clusters when bound to dynamic microtubules while it is monomeric in solution**.......1**

Appendix Table S1: Effect of 100nM anillin on microtubule dynamics**.......2**

Appendix figure S2: Microtubule crosslinking by anillin is polarity independent**....... 3**

Appendix figure S3: Alpha fold prediction of the structure of human anillin**....... 4**

Appendix methods....... 5

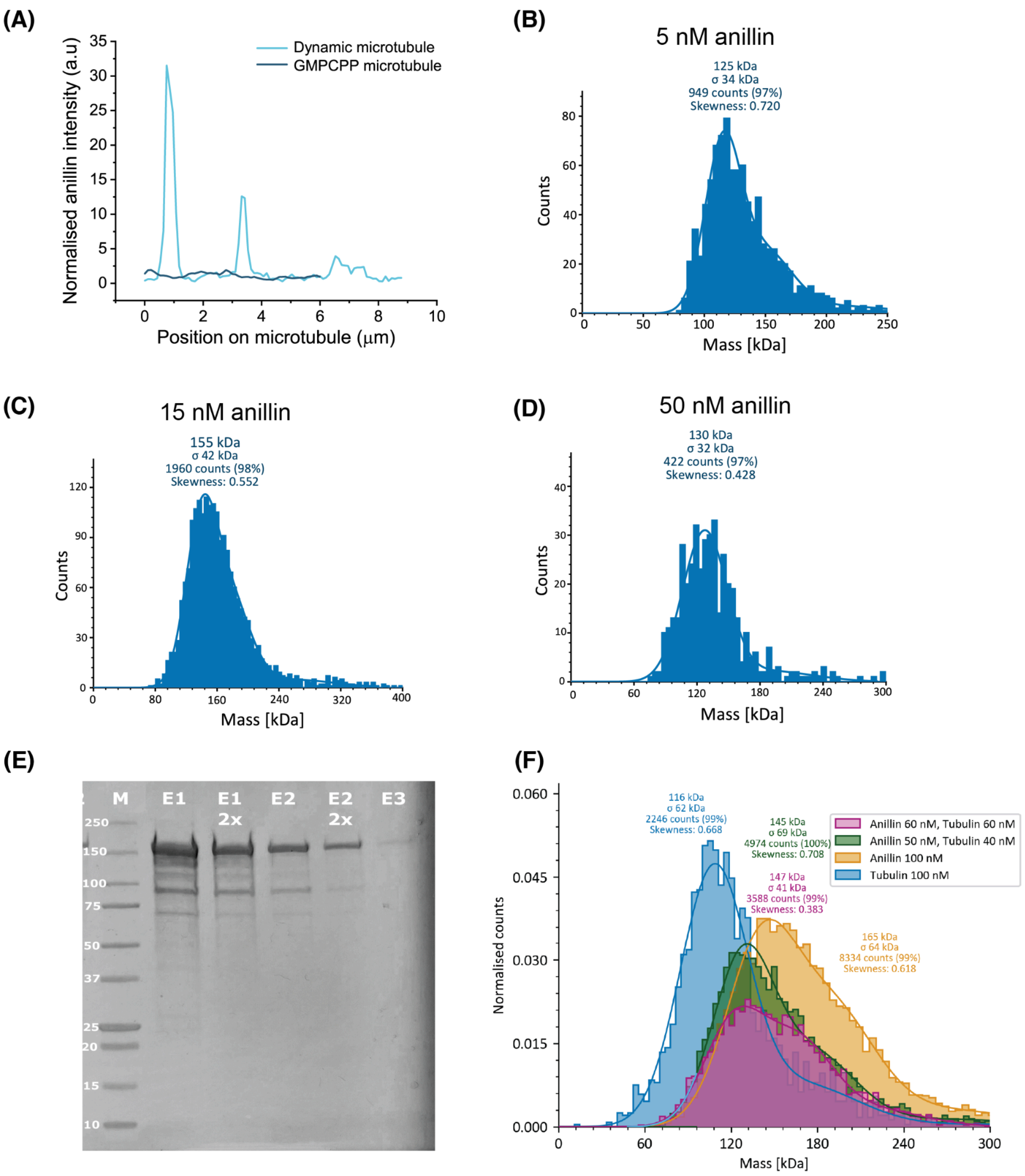

(A)
Dynamic microtubule
GMPCPP microtubule
Normalised anillin intensity (a.u)
Position on microtubule (μm)
(B)
5 nM anillin
125 kDa
σ 34 kDa
949 counts (97%)
Skewness: 0.720
Counts
Mass [kDa]
(C)
15 nM anillin
155 kDa
σ 42 kDa
1960 counts (98%)
Skewness: 0.552
Counts
Mass [kDa]
(D)
50 nM anillin
130 kDa
σ 32 kDa
422 counts (97%)
Skewness: 0.428
Counts
Mass [kDa]
(E)
M
E1
E1 2x
E2
E2 2x
E3
250
150
100
75
50
37
25
20
15
10
(F)
116 kDa
σ 62 kDa
2246 counts (99%)
Skewness: 0.668
145 kDa
σ 69 kDa
4974 counts (100%)
Skewness: 0.708
147 kDa
σ 41 kDa
3588 counts (99%)
Skewness: 0.383
165 kDa
σ 64 kDa
8334 counts (99%)
Skewness: 0.618
Anillin 60 nM, Tubulin 60 nM
Anillin 50 nM, Tubulin 40 nM
Anillin 100 nM
Tubulin 100 nM
Normalised counts
Mass [kDa]

Appendix figure S1: Anillin forms clusters when bound to dynamic microtubules while it is monomeric in solution. (A) Fluorescence intensity profiles for 100 nM anillin on a GMPCPP-stabilized microtubule (kymograph from supplementary figure 1A) versus a dynamic microtubule (kymograph from figure 2F). The intensities were normalised by the median (background-subtracted) intensity. (B), (C) and (D) Mass histograms obtained by iSCAT for anillin-GFP at concentrations of 5, 15 and 50 nM, respectively, showing a main peak corresponding to the expected monomer molecular weight (~ 150 kDa). (E) SDS-PAGE showing purity of anillin (M= marker). (F) Mass histograms obtained by iSCAT for 100 nM anillin-GFP and 100 nM tubulin separately (yellow and blue, respectively) and mixed together (green and pink of 2 different concentration ratios). Note that the expected weight of the tubulin dimer is ~ 110 kDa.

	**Growth speeds**		**Shrinkage speeds**		**Catastrophes**		**Rescues**	
	Time weighted speed (µm/min)	n	Time weighted speed (µm/min)	n	Frequency (min^{-1})	n	Frequency (min^{-1})	n
**Control**	0.92±0.22	186	27.54±8.6	113	0.21±0.02	134	1 observation in 15.83 mins	1
**100 nM anillin (end-accumulated)**	0.94±0.33	32	19.46±7.8	69	1.6±0.16	99	5.1±0.72	50
**100 nM anillin (non-end-accumulated)**	1.13±0.29	124	21.16±11.6	48	0.14±0.02	44	4.5±0.8	32

Appendix Table S1: Effect of 100nM anillin on microtubule dynamics. The growth and shrinkage speeds for dynamic microtubules without (control) and with anillin were obtained from kymographs (n = number of slopes of growth and shrinkage phases analyzed). Speeds measured with anillin were analyzed separately for microtubules with end-accumulated or non-end-accumulated anillin. The error is the weighted standard deviation. Catastrophe frequencies were obtained by dividing the total number of catastrophes (n) with the total time microtubules spent growing. Rescue frequencies were obtained by dividing the total number of rescues (n) with the total time microtubules spent shrinking. The errors in the catastrophe and rescue frequencies are the frequency divided by the square root of the number of events.

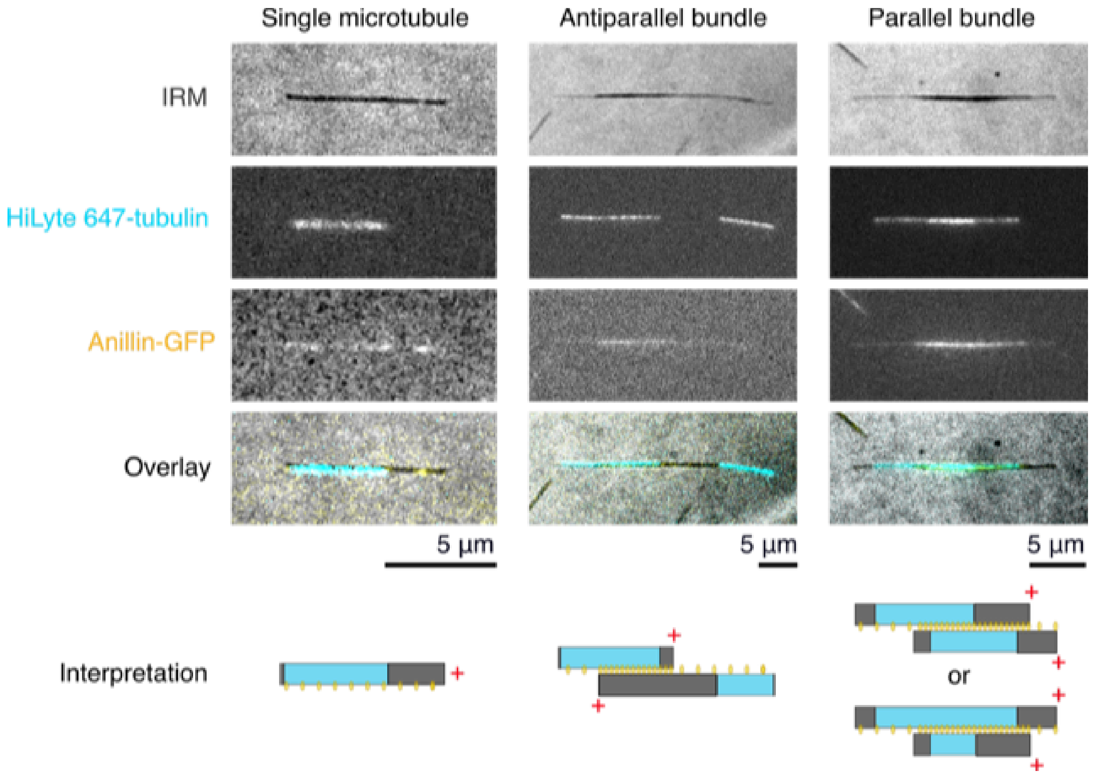

Appendix figure S2: Microtubule crosslinking by anillin is polarity independent. Representative micrographs of an individual microtubule labelled for polarity (left column), antiparallel (middle column), and parallel (right column) microtubule bundles. Cartoon schemes at the bottom represent the interpretation of the images, with a + sign showing the microtubule plus-end.

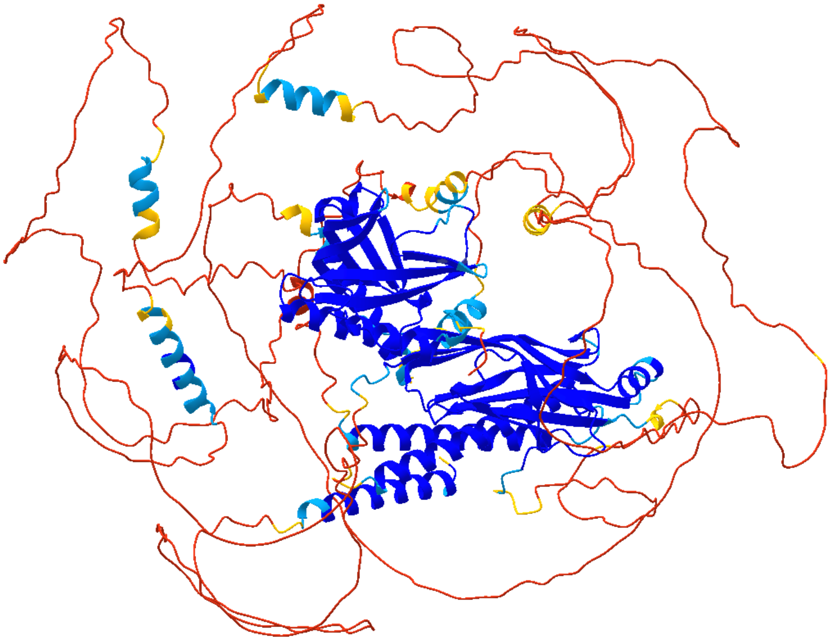

Appendix figure S3: Alpha fold prediction of the structure of human anillin (isoform 2). Unstructured regions (red) are highly prevalent.

Appendix Methods

Mass photometry measurements on anillin and tubulin solutions

Coverslips (Marienfield, 24×50 mm, No. 1.5H) were washed by sonicating first in MilliQ for 5 min, then in 50 % isopropanol for 5 min, and finally in MilliQ for 5 min. Coverslips were then dried using nitrogen gas. To assemble the sample chamber, either 4 or 6 CultureWell gaskets (Grace Biolabs) were cut and then placed on the coverslip. The assembled device was placed on the objective of the Refeyn OneMP Mass Photometer and anillin was diluted in MRB80 to final concentrations in the range of 5-100 nM and injected in the gaskets. The samples were imaged using a 525 nm laser and videos were acquired at a frequency of 300 fps for 6000 frames. To visualise the videos and obtain the mass distribution histograms, the Refeyn DiscoverMP software was used.

Alternately, a coverslip was placed directly on the objective of the Refeyn OneMP Mass Photometer. The instrument was focused using a 9 µL droplet of MRB80 buffer, and 1 µL of anillin or tubulin or a mix of both was then pipetted into the droplet to final concentrations between 30 and 100 nM. The instrument was calibrated using NativeMark™ Unstained Protein Standard (Invitrogen™) to determine molecular weights in kDa.

Assay for determining the polarity-dependence of microtubule crosslinking

First, we elongated HiLyte647-labelled microtubules with unlabelled tubulin. Using interference reflection microscopy (IRM) combined with TIRF microscopy, we then observed the microtubules in a passivated channel in imaging buffer without surface functionalisation, employing a crowding agent (0.3% methyl cellulose) to bring the microtubules to the coverslips. By analysing the relative position of the fluorescence-labelled part of the microtubules, we resolved the structural polarity of these filaments. Next, we verified that at this concentration, methyl cellulose does not promote the formation of stable microtubule bundles. Finally, we introduced anillin-GFP to a final concentration of approximately 500 nM into the channel. Upon the introduction of anillin, we began observing the formation of microtubule bundles. From these, we selected those in which we could resolve the structural polarity of the constituent microtubules.

Alpha fold prediction:

The predicted three-dimensional structure of Anillin was generated with AlphaFold3 (DeepMind, 2024). The top-ranked model was visualized using PyMOL, with residues colored according to the predicted B-value (confidence) spectrum, where blue indicates high-confidence regions and red indicates low-confidence regions.
