# Supplementary material for: Anillin directly crosslinks microtubules with actin filaments
Source: EMBO J. 2025 Jul 21;44(17):4803–24. doi: 10.1038/s44318-025-00492-3 (PMC12402178; doi:10.1038/s44318-025-00492-3)
Supplement: Supplementary file 3 — Movie EV1 [file 44318_2025_492_MOESM3_ESM.zip › Movie EV1/Movie EV1 legend.docx]

**Movie EV1:** GMPCPP microtubules zippering together after flowing in anillin (12 nM) at t=0
