# Supplementary material for: Anillin directly crosslinks microtubules with actin filaments
Source: EMBO J. 2025 Jul 21;44(17):4803–24. doi: 10.1038/s44318-025-00492-3 (PMC12402178; doi:10.1038/s44318-025-00492-3)
Supplement: Supplementary file 4 — Movie EV2 [file 44318_2025_492_MOESM4_ESM.zip › Movie EV2/Movie EV2 legend.docx]

**Movie EV2:** Detachment and breaking of a GMPCPP microtubule while being crosslinked to another GMPCPP microtubule upon addition of 12 nM anillin.
