# Supplementary material for: Anillin directly crosslinks microtubules with actin filaments
Source: EMBO J. 2025 Jul 21;44(17):4803–24. doi: 10.1038/s44318-025-00492-3 (PMC12402178; doi:10.1038/s44318-025-00492-3)
Supplement: Supplementary file 5 — Movie EV3 [file 44318_2025_492_MOESM5_ESM.zip › Movie EV3/Movie EV3 legend.docx]

**Movie EV3:** Short actin filaments (red) getting recruited to a long GMPCPP microtubule (cyan) by anillin (yellow) and diffusing and sliding over it.
