# Supplementary material for: Anillin directly crosslinks microtubules with actin filaments
Source: EMBO J. 2025 Jul 21;44(17):4803–24. doi: 10.1038/s44318-025-00492-3 (PMC12402178; doi:10.1038/s44318-025-00492-3)
Supplement: Supplementary file 6 — Movie EV4 [file 44318_2025_492_MOESM6_ESM.zip › Movie EV4/Movie EV4 legend.docx]

**Movie EV4:** Actin filaments (red) bind and unbind from a dynamic microtubule (cyan) which has anillin (yellow) diffusing on it. Scale bar = 2 µm
