# Supplementary material for: Anillin directly crosslinks microtubules with actin filaments
Source: EMBO J. 2025 Jul 21;44(17):4803–24. doi: 10.1038/s44318-025-00492-3 (PMC12402178; doi:10.1038/s44318-025-00492-3)
Supplement: Supplementary file 7 — Movie EV5 [file 44318_2025_492_MOESM7_ESM.zip › Movie EV5/Movie EV5 legend.docx]

**Movie EV5:** Anillin (yellow) facilitating the diffusion of an actin filament (red) on the microtubule (cyan). Scale bar = 2 µm
