# Supplementary material for: Anillin directly crosslinks microtubules with actin filaments
Source: EMBO J. 2025 Jul 21;44(17):4803–24. doi: 10.1038/s44318-025-00492-3 (PMC12402178; doi:10.1038/s44318-025-00492-3)
Supplement: Supplementary file 8 — Movie EV6 [file 44318_2025_492_MOESM8_ESM.zip › Movie EV6/Movie EV6 legend.docx]

**Movie EV6**: Actin filament (red) captured on the microtubule (cyan) + end by anillin (yellow) and then moving along with the growing and shrinking end. Scale bar = 2 µm
