# Supplementary material for: Anillin directly crosslinks microtubules with actin filaments
Source: EMBO J. 2025 Jul 21;44(17):4803–24. doi: 10.1038/s44318-025-00492-3 (PMC12402178; doi:10.1038/s44318-025-00492-3)
Supplement: Supplementary file 9 — Movie EV7 [file 44318_2025_492_MOESM9_ESM.zip › Movie EV7/Movie EV7_legend.docx]

**Movie EV7:** Multiple actin filaments (red) getting captured on a dynamic microtubule (cyan) due to anillin (yellow). Note that in this example eventually the bundle also moves with the shrinking microtubule. Scale bar = 2 µm
