# Supplementary figures and images for: Anillin directly crosslinks microtubules with actin filaments
Source: EMBO J. 2025 Jul 21;44(17):4803–24. doi: 10.1038/s44318-025-00492-3 (PMC12402178; doi:10.1038/s44318-025-00492-3)

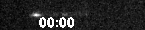

Supplement: Supplementary file 10 — Source data Fig. 1 [file 44318_2025_492_MOESM10_ESM.zip › Figure 1/1H/100nManillin.tif]

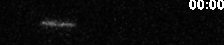

Supplement: Supplementary file 10 — Source data Fig. 1 [file 44318_2025_492_MOESM10_ESM.zip › Figure 1/1F/30nManillin.tif]

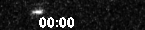

Supplement: Supplementary file 10 — Source data Fig. 1 [file 44318_2025_492_MOESM10_ESM.zip › Figure 1/1G/50nManillin.tif]

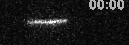

Supplement: Supplementary file 10 — Source data Fig. 1 [file 44318_2025_492_MOESM10_ESM.zip › Figure 1/1E/15nManillin.tif]

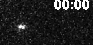

Supplement: Supplementary file 10 — Source data Fig. 1 [file 44318_2025_492_MOESM10_ESM.zip › Figure 1/1D/1nManillin.tif]

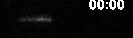

Supplement: Supplementary file 10 — Source data Fig. 1 [file 44318_2025_492_MOESM10_ESM.zip › Figure 1/1C/Control_0nManillin.tif]

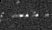

Supplement: Supplementary file 11 — Source data Fig. 2 [file 44318_2025_492_MOESM11_ESM.zip › Figure 2/2A/Biot_nonbiot_GMPCPPMT_overlap.tif]

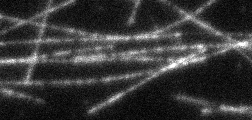

Supplement: Supplementary file 11 — Source data Fig. 2 [file 44318_2025_492_MOESM11_ESM.zip › Figure 2/2B/MTbundling_left.tif]

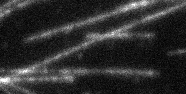

Supplement: Supplementary file 11 — Source data Fig. 2 [file 44318_2025_492_MOESM11_ESM.zip › Figure 2/2B/MTBundling_Right.tif]

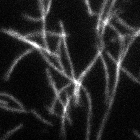

Supplement: Supplementary file 12 — Source data Fig. 3 [file 44318_2025_492_MOESM12_ESM.zip › Figure 3/3B/actin_bundle_5nManillin.tif]

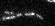

Supplement: Supplementary file 12 — Source data Fig. 3 [file 44318_2025_492_MOESM12_ESM.zip › Figure 3/3A/actin_filament_10nManillin.tif]

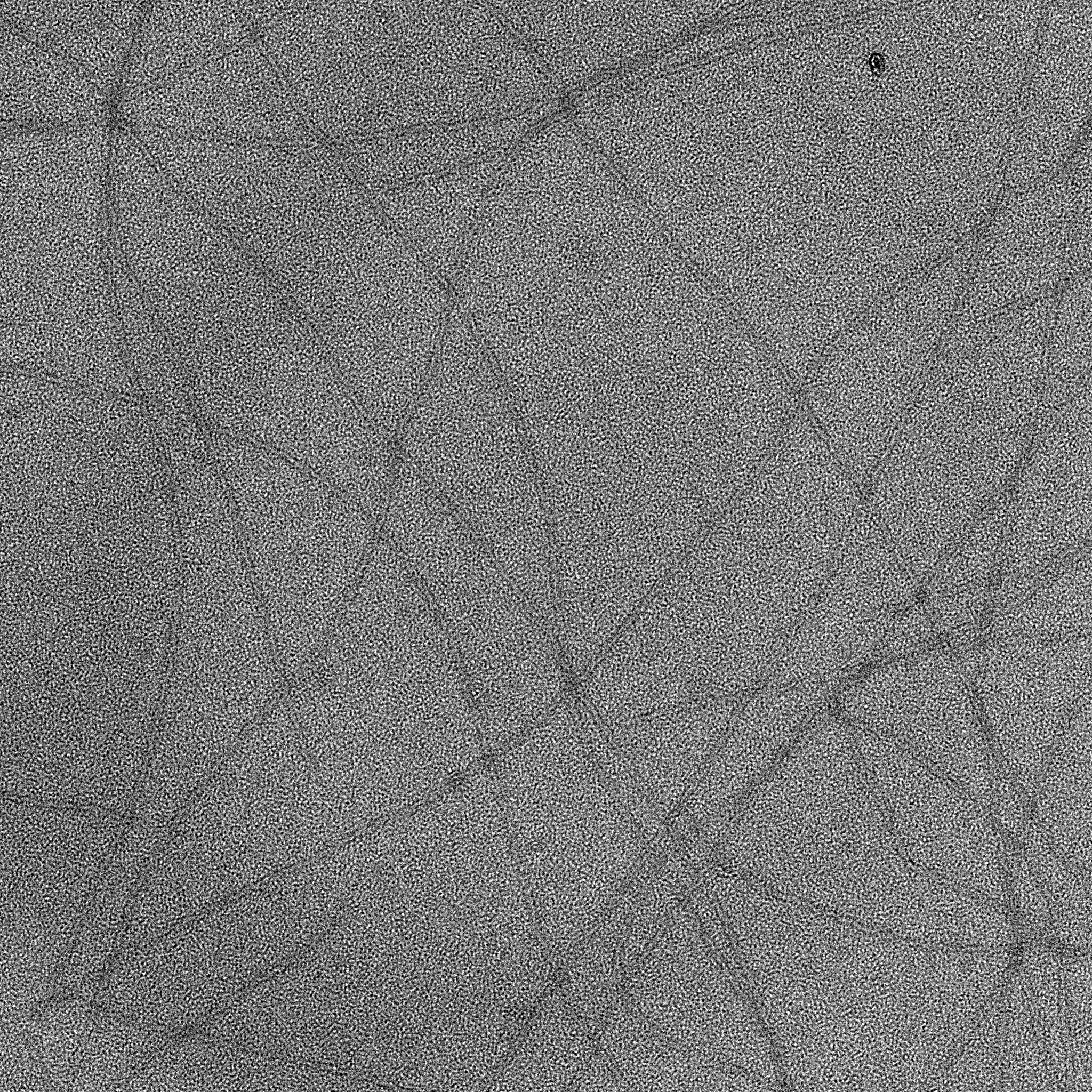

Supplement: Supplementary file 12 — Source data Fig. 3 [file 44318_2025_492_MOESM12_ESM.zip › Figure 3/3F/Actin_control.tif]

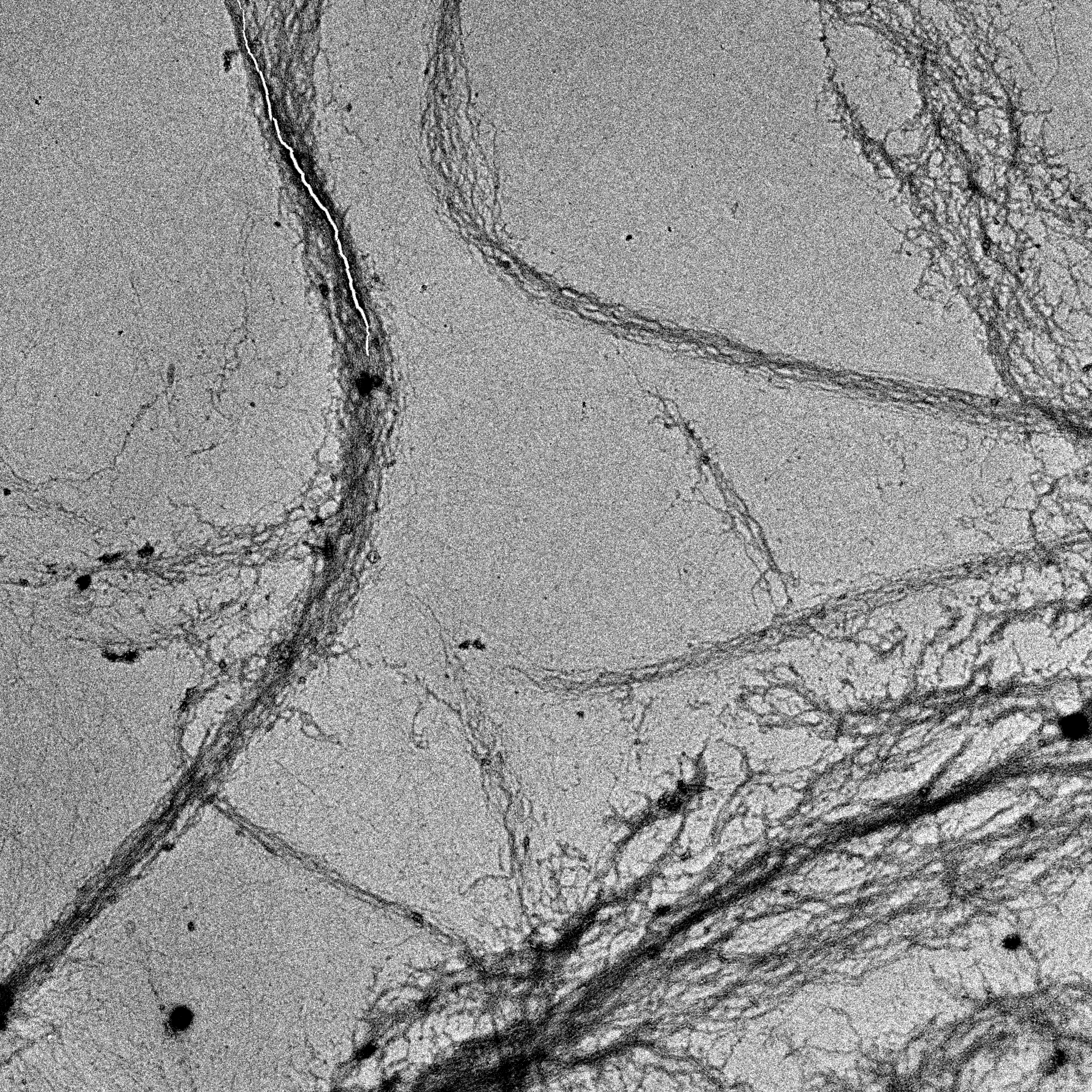

Supplement: Supplementary file 12 — Source data Fig. 3 [file 44318_2025_492_MOESM12_ESM.zip › Figure 3/3F/Actin_bundle_left.tif]

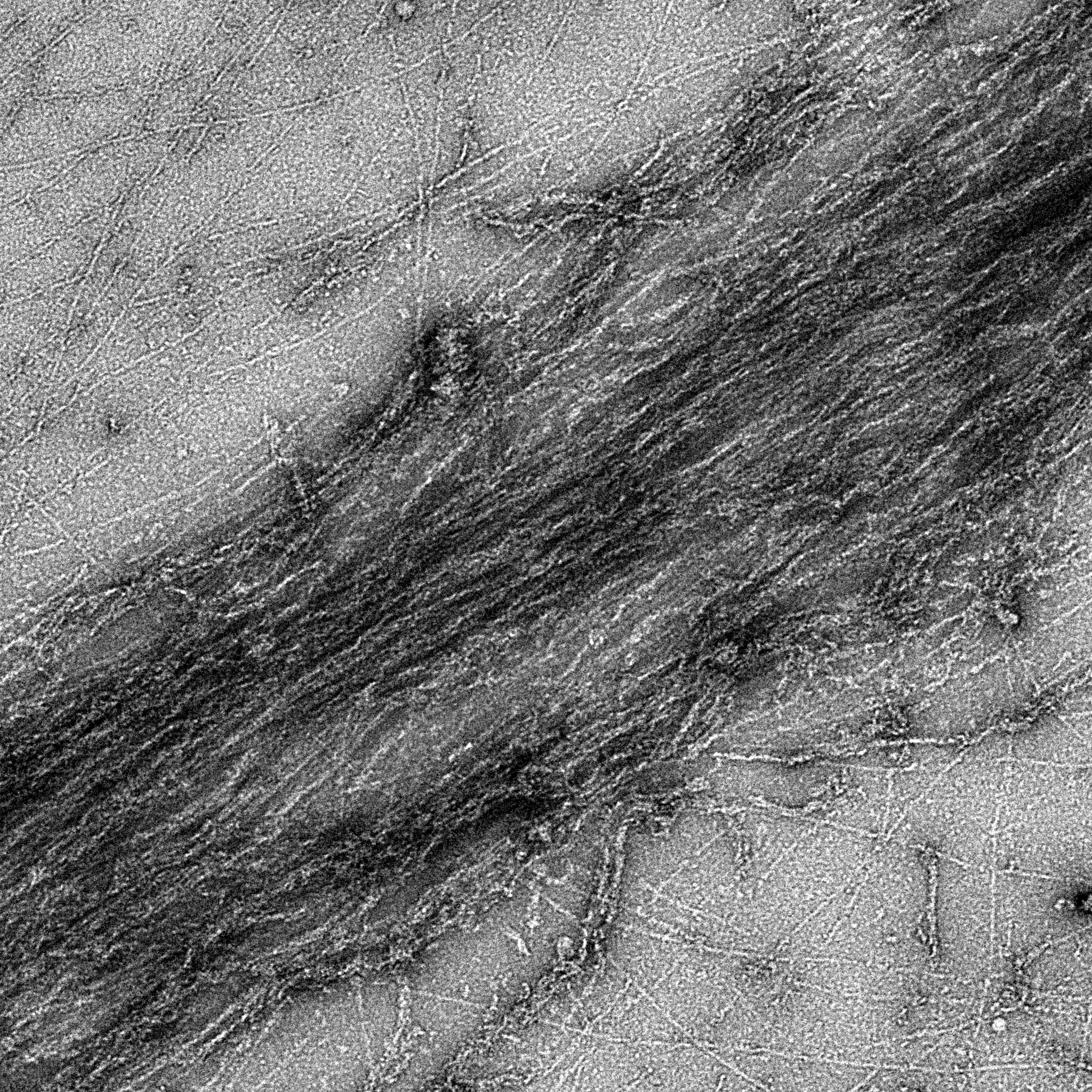

Supplement: Supplementary file 12 — Source data Fig. 3 [file 44318_2025_492_MOESM12_ESM.zip › Figure 3/3F/Actin_bundle_right.tif]

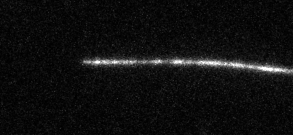

Supplement: Supplementary file 13 — Source data Fig. 4 [file 44318_2025_492_MOESM13_ESM.zip › Figure 4/4B/Actin_sliding.tif]

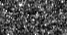

Supplement: Supplementary file 13 — Source data Fig. 4 [file 44318_2025_492_MOESM13_ESM.zip › Figure 4/4C/i_10nManillin_intensity_increase.tif]

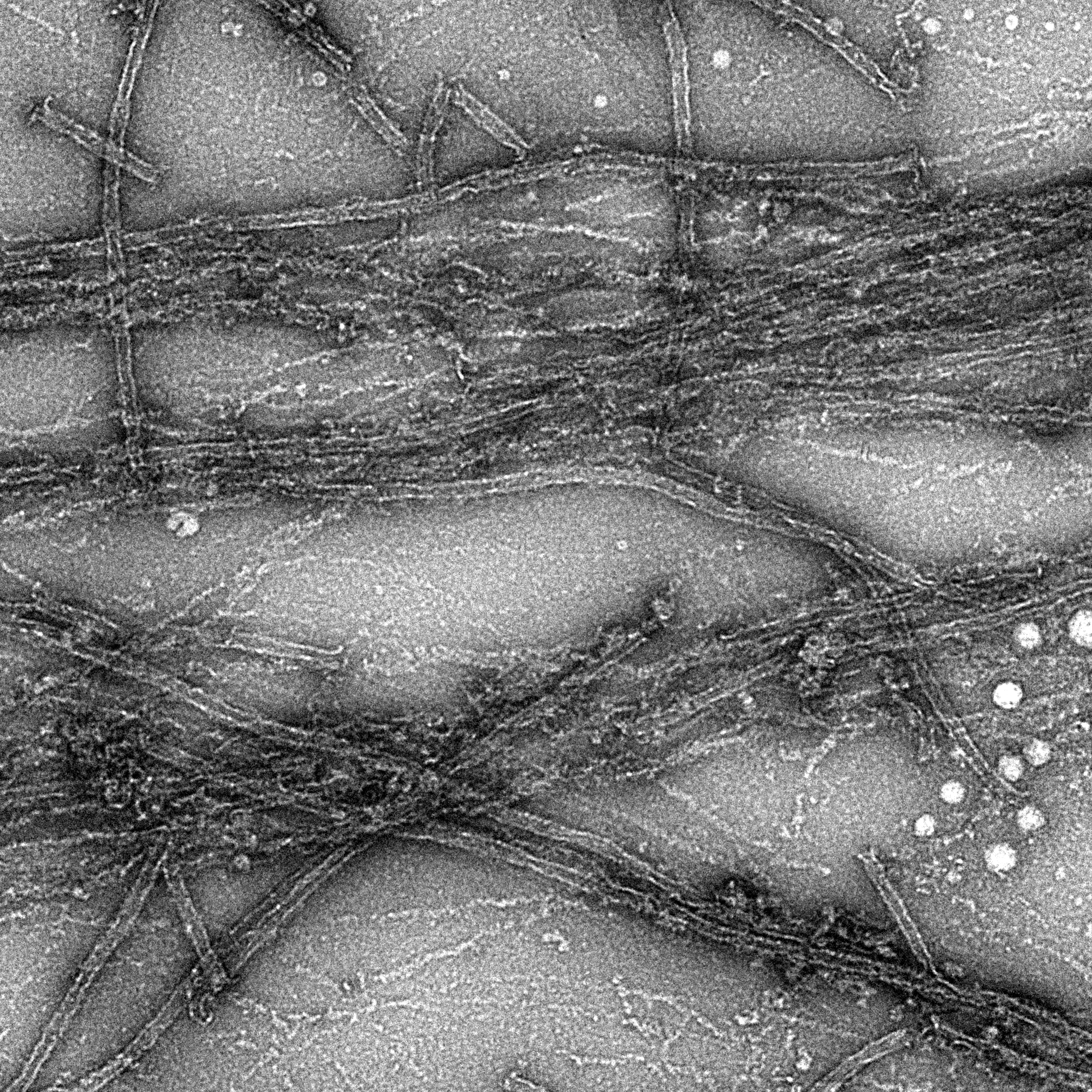

Supplement: Supplementary file 13 — Source data Fig. 4 [file 44318_2025_492_MOESM13_ESM.zip › Figure 4/4D/iii_act_MT_anillin.tif]

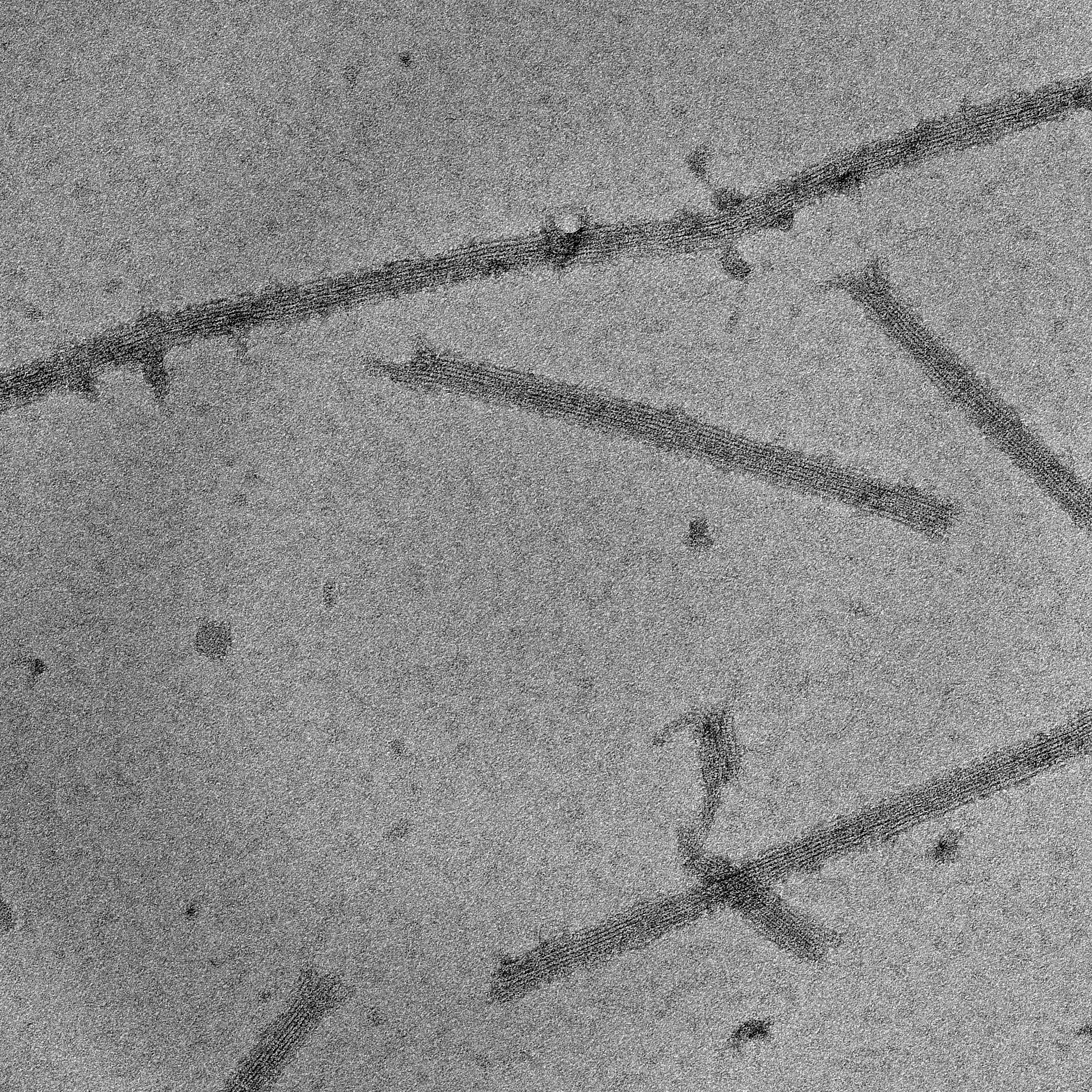

Supplement: Supplementary file 13 — Source data Fig. 4 [file 44318_2025_492_MOESM13_ESM.zip › Figure 4/4D/i_anillin_MT_control.tif]

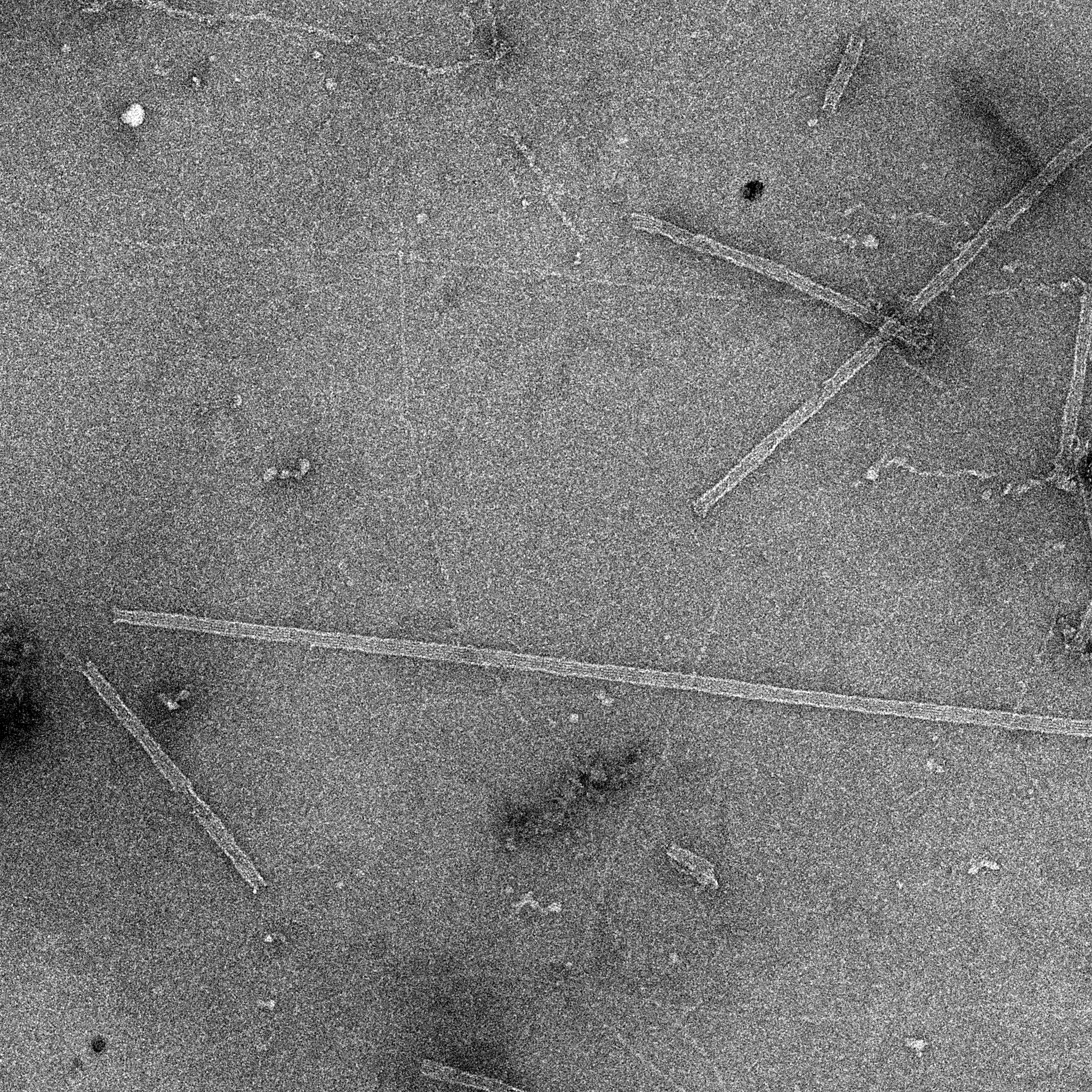

Supplement: Supplementary file 13 — Source data Fig. 4 [file 44318_2025_492_MOESM13_ESM.zip › Figure 4/4D/ii_act_MT_control.tif]

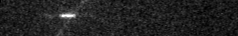

Supplement: Supplementary file 14 — Source data Fig. 5 [file 44318_2025_492_MOESM14_ESM.zip › Figure 5/5A/Binding_unbinding.tif]

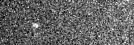

Supplement: Supplementary file 14 — Source data Fig. 5 [file 44318_2025_492_MOESM14_ESM.zip › Figure 5/5C/Tip_tracking.tif]

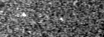

Supplement: Supplementary file 14 — Source data Fig. 5 [file 44318_2025_492_MOESM14_ESM.zip › Figure 5/5D/Bundling.tif]

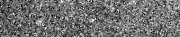

Supplement: Supplementary file 14 — Source data Fig. 5 [file 44318_2025_492_MOESM14_ESM.zip › Figure 5/5B/Lattice_sliding.tif]
