# Supplementary material for: Anillin directly crosslinks microtubules with actin filaments
Source: EMBO J. 2025 Jul 21;44(17):4803–24. doi: 10.1038/s44318-025-00492-3 (PMC12402178; doi:10.1038/s44318-025-00492-3)
Supplement: Supplementary file 15 — Expanded View Figures [file 44318_2025_492_MOESM15_ESM.pdf]

Expanded View Figures

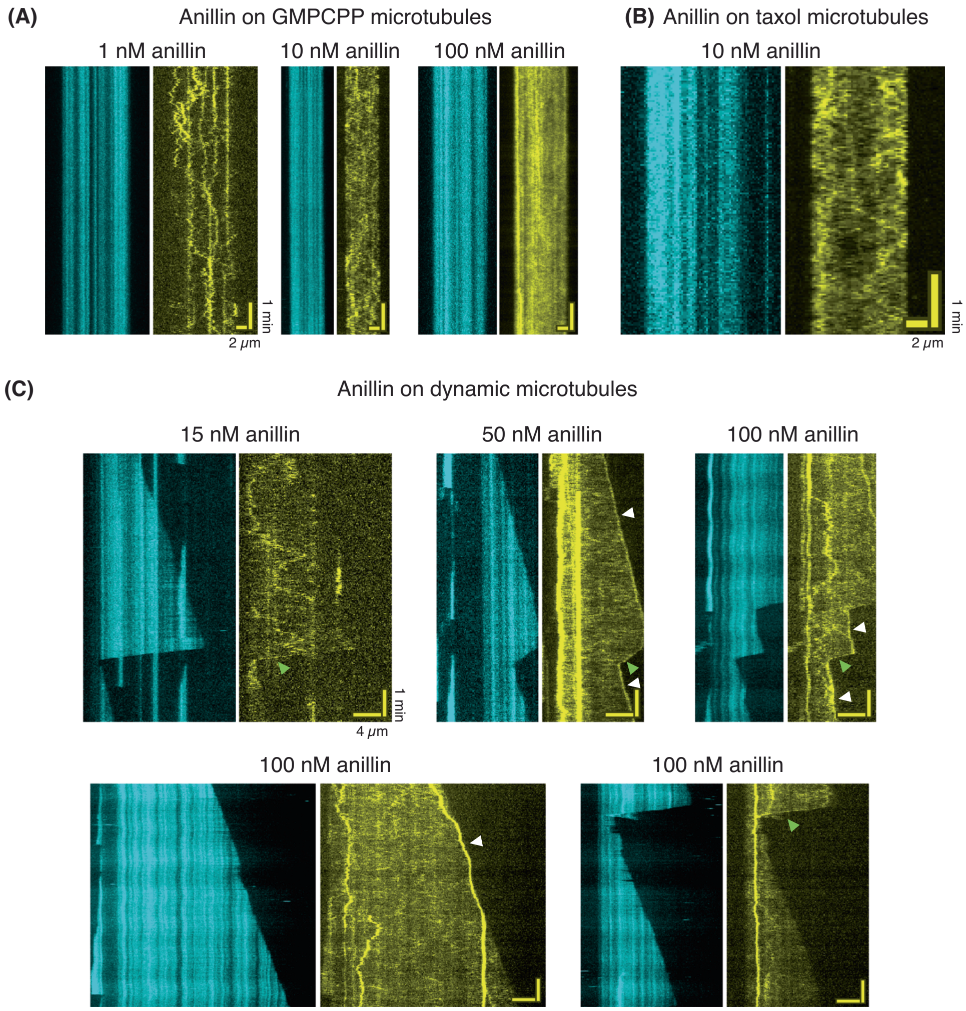

Figure EV1. Anillin binds to microtubules.

(**A**) Kymographs of anillin-GFP (yellow) diffusing on GMPCPP microtubules (cyan) at different concentrations (see legend). (**B**) Kymograph of 10 nM anillin-GFP (yellow) diffusing on a taxol microtubule (cyan). (**C**) Example kymographs of anillin-GFP (yellow) diffusing on dynamic microtubules (cyan) and tracking their + ends at different concentrations (see legend). White arrowheads point to the accumulation of anillin on growing microtubule ends, whereas green arrowheads point to accumulations of anillin on shrinking microtubule ends. Vertical axis denotes time, horizontal axis denotes space (see legends for scale bars).

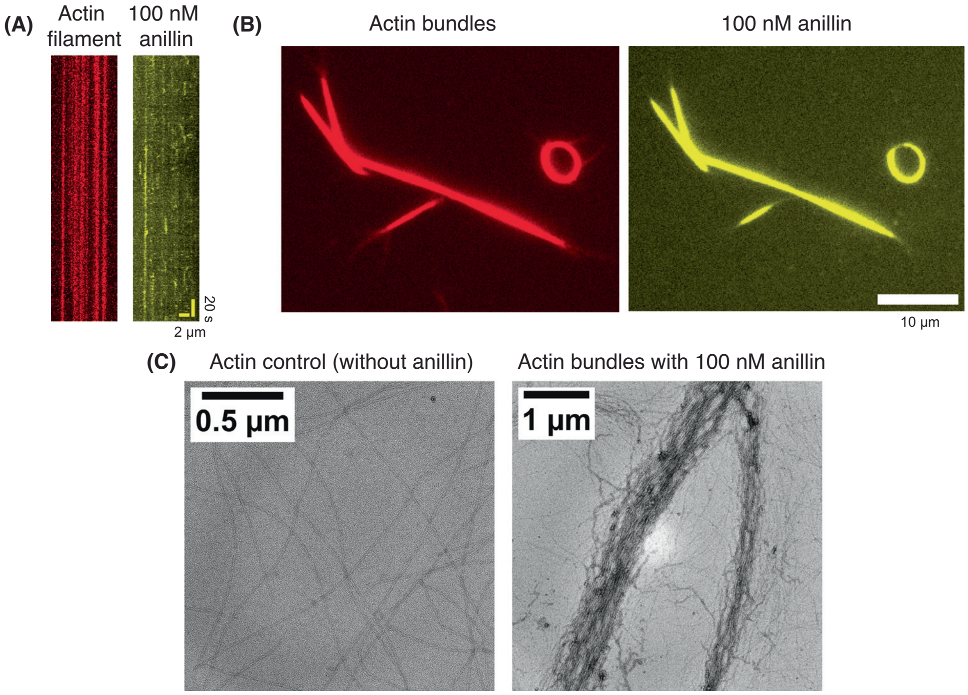

Figure EV2. Anillin forms heterogenous and loose actin bundles.

(**A**) Kymograph of 100 nM anillin (yellow) bound to a single surface-immobilised actin filament (red). (**B**) TIRF images of actin bundles formed by 100 nM anillin. (**C**) EM images of actin filaments without (left; re-displayed from Fig. 3F here as a reference) and with 100 nM anillin (right). With anillin, clear bundling of actin filaments is observed, and these bundles are loose with different widths.

 © The Author(s)

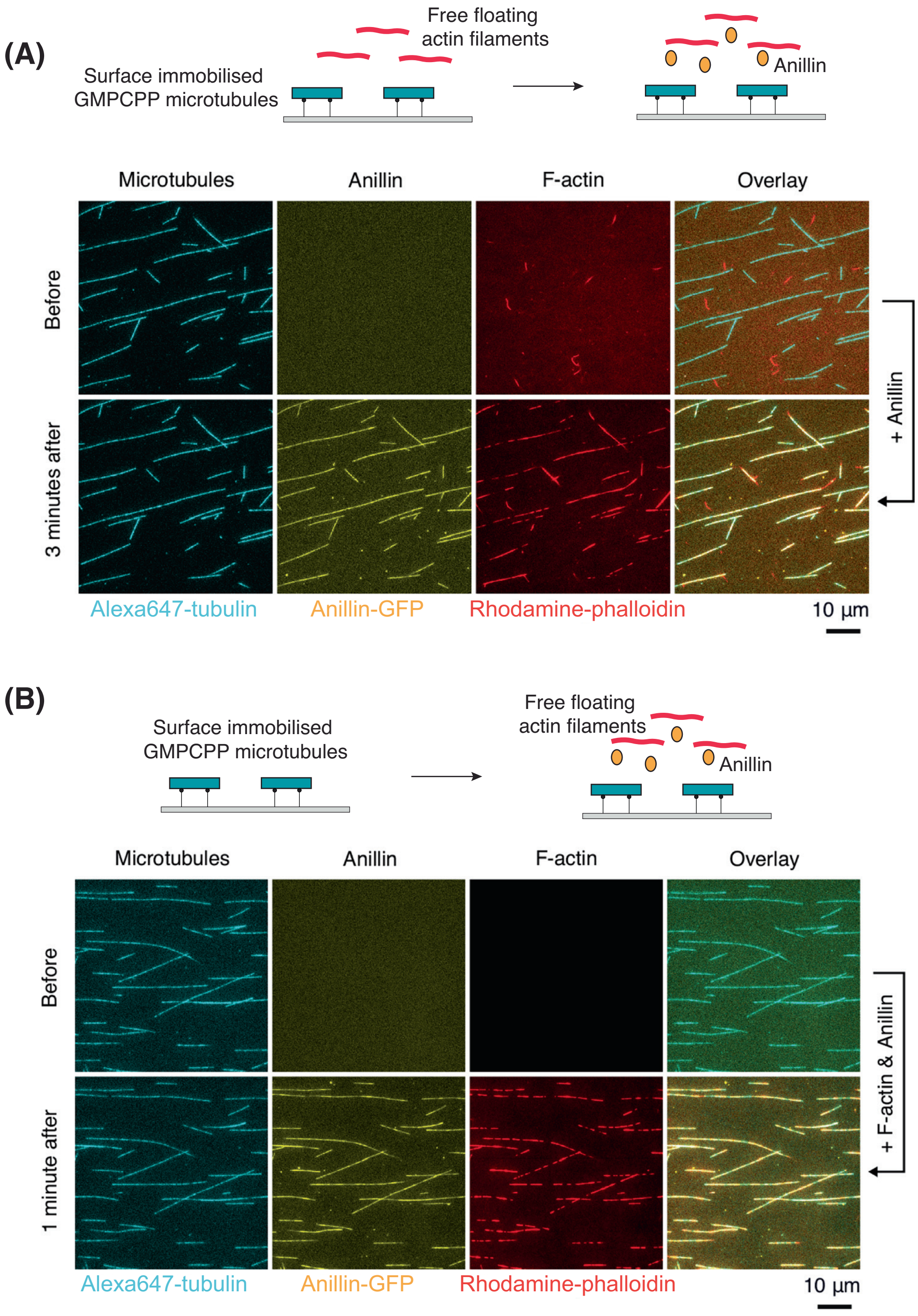

Figure EV3. Crosslinking of actin filaments and microtubules by anillin occurs irrespective of the order in which components are combined.

(**A**) Top: The flow channel initially contains surface-immobilised GMPCPP microtubules (cyan) and a solution of actin filaments (red) without anillin. Under these conditions, actin filaments freely diffuse and do not bind to the microtubules. Bottom: Once anillin-GFP (yellow) is flown in, actin filaments bind to the microtubules. (**B**) Top: The channel contains only surface-immobilised microtubules (cyan). Bottom: A solution of anillin-GFP (yellow) and actin filaments (red) is flown in, causing actin filaments to get bound to the microtubules. Sale bars 10 µm.

© The Author(s)

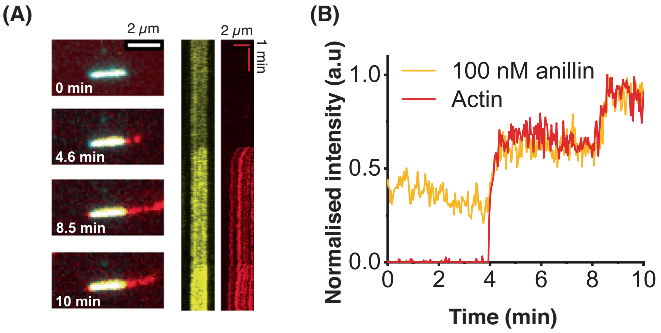

Figure EV4. Anillin crosslinks actin filaments and microtubules.

Increase of the intensity of anillin (100 nM) upon the recruitment of actin filaments on a GMPCPP microtubule. The still images and kymograph (**A**) and corresponding fluorescence intensity plot (**B**) show that the anillin signal increases simultaneously with the recruitment of actin filaments.

© The Author(s)
